# Surveillance of Swine Coronaviruses in Hungarian Herds with a Newly Established Pan-Coronavirus RT-PCR System

**DOI:** 10.3390/ani16030358

**Published:** 2026-01-23

**Authors:** Dóra Máté, Renáta Varga-Kugler, Eszter Kaszab, Henrik Fülöp Károlyi, Tamás Görföl, Gábor Kemenesi, Barbara Igriczi, Gyula Balka, Marianna Domán, Ádám Bálint, Zoltán Zádori, Enikő Fehér

**Affiliations:** 1 Department of Microbiology and Infectious Diseases, University of Veterinary Medicine Budapest, Hungária krt. 23-25, H-1143 Budapest, Hungary; 2 Ceva-Phylaxia Ltd., Szállás utca 5, H-1107 Budapest, Hungary; 3 National Laboratory for Infectious Animal Diseases, Antimicrobial Resistance, Veterinary Public Health and Food Chain Safety, István utca 2, H-1078 Budapest, Hungary; 4 Department of Bioinformatics, One Health Institute, Faculty of Health Sciences, University of Debrecen, Nagyerdei krt. 98, H-4032 Debrecen, Hungary; 5 National Laboratory of Virology, Szentágothai Research Centre, University of Pécs, Ifjúság útja 20, H-7624 Pécs, Hungary; 6 Institute of Biology, Faculty of Sciences, University of Pécs, Ifjúság útja 6, H-7624 Pécs, Hungary; 7 Department of Pathology, University of Veterinary Medicine Budapest, István utca 2, H-1078 Budapest, Hungary; 8 HUN-REN Veterinary Medical Research Institute, Hungária krt. 21, H-1143 Budapest, Hungary; 9 Vetcontrol Ltd., Déli-Bekötő út 8, H-1211 Budapest, Hungary

**Keywords:** pan-coronavirus, PCR, mammalian, avian, human, swine

## Abstract

The rapid evolution of coronaviruses (CoVs) requires researchers to develop specific yet broad-spectrum detection methods to monitor their constant genomic changes. The goal of the present study was to establish a current pan-coronavirus RT-PCR system capable of detecting a wide variety of CoVs and useful for the investigation of virus diversity and host spectrum. For optimization, one-step and two-step nested RT-PCRs with three RT enzymes were examined, amplifying a ~600 bp long product of the RNA-dependent RNA polymerase. As templates, the in vitro transcribed RNA of ten pathogenic CoVs (SARS-CoV, SARS-CoV-2, NL-63, OC43, feline CoV, porcine epidemic diarrhea virus or PEDV, transmissible gastroenteritis virus or TGEV, canine CoV, bat CoV, and infectious bronchitis virus) were applied instead of the often-used DNA standards. A limit of detection of 5–50 copies/reaction was achieved with a random hexamer-primed two-step RT-PCR and a touchdown cycling profile, representing a lower detection limit and higher specificity compared to previously published primer sets. Swine origin pooled samples (*n* = 121), collected from apparently healthy herds in Hungary, were tested with the novel RT-PCR system. Sequences of porcine respiratory CoV/TGEV and porcine hemagglutinating encephalomyelitis virus were identified in 24 oral fluid and nasal swab pools, demonstrating the circulation of these viruses in this country, as well as the suitability of the new PCR for their detection. The results highlighted the importance of adequate RT enzyme selection and the use of RNase inhibitors in sample preparation and conservation.

## Simple Summary

Due to the SARS-CoV-2 pandemic, we have become familiar with coronaviruses. These viruses have a highly diverse, rapidly changing genome that aids in widespread distribution and transmission to novel hosts. Although several simple, virus-specific tests are available to detect the most important pathogenic coronaviruses, the identification of new viruses and variants requires different approaches. In our study, we developed a broad-spectrum detection system for coronaviruses. The sensitivity of the assay was measured using ten different coronaviruses that infect humans and animals and was compared with other systems. Application of the novel method demonstrated the circulation of coronaviruses in swine herds, as well as the suitability of the established tool for their detection. The assay described here is primarily intended for research purposes and could significantly advance our understanding of the diversity and host spectrum of coronaviruses and help us prepare for the emergence of new pathogens.

## 1. Introduction

Most people associate the word coronavirus (CoV) with the disease acronym COVID-19 and the causative agent behind it, severe acute respiratory syndrome CoV-2 (SARS-CoV-2). However, CoVs of veterinary importance are also widespread. Genome and phylogenetic analyses have revealed the complex and devious evolution of CoVs. A well-known phenomenon is the propensity for recombination in cases of co-infections of multiple CoVs. Recombination and accumulation of mutations, as consequences of imprecise nucleotide incorporations driven by the RNA-dependent RNA polymerase (RdRp), allow for high adaptability, resulting in the emergence and spread of new viruses, as well as cross-species transmission. Spillover events might lead to zoonosis and implicate the possibility of reverse zoonotic infections among humans and mammals [1,2,3,4,5,6,7].

CoVs (family *Coronaviridae*, order *Nidovirales*) are classified into the *Alpha*-, *Beta*-, *Gamma*-, and *Deltacoronavirus* genera [8]. The *Alphacoronavirus* and *Betacoronavirus* genera include mammalian- and human-associated viruses, while the *Gamma*- and *Deltacoronavirus* genera contain viruses infecting both mammals and birds [8]. The virions are enveloped and have a 25–31 kb long, positive-sense, single-stranded RNA genome. Their genomic structure is highly diverse; open reading frames (ORF) 1a and 1b of the non-structural proteins, as well as the ORFs of the nucleocapsid (N), spike (S), membrane (M), and envelope (E) proteins, are found and arranged in this order, while the number and function of the accessory protein-encoding genes vary even among strains [9]. The much-studied and common diagnostic target S protein shows high variability among species. It mediates the receptor binding, the entry of the virus into the cell, and the activation of the immune system [9].

CoVs have been blamed for diseases of various types and severity. Humans are often encountered with common cold-like conditions caused by HCoV-229E, HCoV-NL63, HCoV-OC43, or HCoV-HKU1 [10]. The most notorious, human-associated, zoonotic CoVs (SARS-CoV, SARS-CoV-2, and Middle East respiratory syndrome CoV) are responsible for systemic, even fatal, syndromes [10,11,12,13]. Regarding veterinary aspects of CoV infections, several economically important CoVs can be identified, including the infectious bronchitis virus (IBV), bovine CoV, feline CoV (FCoV), canine CoVs (CCoV), and swine CoVs (porcine epidemic diarrhea virus or PEDV; transmissible gastroenteritis virus or TGEV; porcine respiratory CoV or PRCV, a deletion variant of TGEV; porcine hemagglutinating encephalomyelitis virus or PHEV; swine acute diarrhea syndrome CoV; porcine delta CoV) [1,4,5,14,15,16,17,18,19,20,21]. Due to their role in CoV evolution, monitoring bat CoVs is definitely important [4,22]. Early recognition of CoV infections and emerging variants has a significant impact on human and animal healthcare. However, overall detection is challenging due to their genomic diversity.

In this study, we aimed to develop a universal pan-coronavirus detection system with the broadest possible specificity, primarily for researchers to reveal the diversity and genomic changes of CoV. This method has been successfully tested on human and animal CoV isolates and samples with three different RT enzymes. Field samples from swine were surveyed, and besides PRCV/TGEV, PHEV sequences could be identified. Based on the control and field sample collection, the primers described herein represented higher specificity when compared to previously published oligonucleotides.

## 2. Materials and Methods

### 2.1. Samples

Clinical samples of animal origin (bat, swine, canine, poultry) and coronavirus isolates (FCoV, NL-63, OC43) were provided by the Department of Pathology, University of Veterinary Medicine, Budapest, Hungary; the HUN-REN Veterinary Medical Research Institute, Budapest, Hungary; and the National Laboratory of Virology, Szentágothai Research Centre, University of Pécs, Hungary. Nucleic acid was extracted with the NucleoSpin RNA Virus kit (Macherey-Nagel, Düren, Germany) according to the manufacturer’s instructions. RNAs of SARS-CoV and SARS-CoV-2 variants “alpha”, “delta”, and “omicron” were extracted and supplied by the National Laboratory of Virology, Szentágothai Research Centre, University of Pécs, Hungary, in accordance with the appropriate biosafety requirements.

Altogether, 121 pooled oral fluid (*n* = 84, one sampling rope per pen), nasal swab (*n* = 34, pooled samples of 4–5 swine per sample, from 162 animals), and processing fluid (*n* = 3, retrieved during castration) specimens of clinically healthy swine were tested for CoVs. These were collected between 2020 and 2022 from pig farms in Hungary from 3–7-day-old piglets (processing fluid), 2–10-week-old (nasal swab) and 8–12-week-old (oral fluid) pigs, and 17–30-week-old fatteners (oral fluid) that did not show clinical signs of disease during the sampling period [23,24]. In terms of their type, these were farrow-to-finish farms for industrial raising with variations in genetics and sow herd size (520–2200 sows) [23,24]. Coronavirus vaccines were not applied to the herds where data were available.

Nucleic acid was extracted with the QIAmp cador Pathogen Mini Kit (QIAGEN, Hilden, Germany) using the QIAcube instrument according to the instructions.

### 2.2. RT and PCR

For the primary testing of oligonucleotides, the RT step was carried out with SuperScript IV Reverse Transcriptase (SSIV, Thermo Scientific, Waltham, MA, USA). As a first step, the following components were combined and incubated at 65 °C for 5 min and then put on ice: 7.5 µL of nuclease-free water, 1 µL of 50 µM random hexamer primer mix, 1 µL of 10 mM dNTP mix, and 5 µL of RNA template. As a second step, 4 µL of 5× SSIV Buffer, 0.5 µL of RiboLock RNase Inhibitor (Thermo Scientific, Waltham, MA, USA), and 1 µL of SSIV enzyme were added to reach the final volume of 20 µL, and the mixture was incubated at 23 °C for 10 min, 55 °C for 10 min, and 80 °C for 10 min. SSIV was applied for RT with variable CoV-specific reverse primer sets as well, using 1 µL of 10 µM primer mixtures (Table 1) with an incubation protocol of 55 °C for 10 min and 80 °C for 10 min.

RevertAid Reverse Transcriptase (Thermo Scientific, Waltham, MA, USA) was tested with a protocol corresponding to that described for SSIV. The mixture was incubated at 25 °C for 10 min, 50 °C for 60 min, and 70 °C for 10 min.

Coronavirus sequences were obtained from GenBank and aligned for PCR primer design (File S1). The coronavirus-specific nested PCRs were performed in a 25 μL volume containing 1× DreamTaq Green buffer, 200 μM of dNTP mix, 400 nM of the Fw-mix1 primer, 400 nM of the Rev-mix1/Rev-mix2 primers (for the first and second PCR of the nested protocol, respectively, Table 1), 0.75 U of DreamTaq DNA Polymerase (Thermo Fisher Scientific, Waltham, MA, USA), and 2 μL of the RT reaction mixture. The normal cycling protocol consisted of an initial denaturation step at 95 °C for 5 min, 35/40 cycles (for the first and second PCR of the nested protocol, respectively) of the steps denaturation at 95 °C for 30 s, primer annealing at 53 °C for 30 s, and extension at 72 °C for 1 min, as well as a final extension step at 72 °C for 10 min. A touchdown cycling protocol was executed for the first PCR step of the nested PCRs as follows: initial denaturation step at 95 °C for 3 min; 10 cycles of denaturation at 95 °C for 30 s, primer annealing at 62–53 °C for 30 s with 1 °C decrease per cycle, and extension at 72 °C for 1 min; 30 cycles of denaturation at 95 °C for 30 s, primer annealing at 53 °C for 30 s, and extension at 72 °C for 1 min; a final extension step at 72 °C for 5 min. Following touchdown cycling, the second PCR of the nested protocol corresponded to that described above for the nested PCR.

One-step RT-PCR was carried out with the QIAGEN OneStep RT-PCR Kit (QIAGEN, Hilden, Germany) in a 25 μL volume containing 1× QIAGEN One-Step RT-PCR Buffer, 400 nM of the Fw-mix primer, 600 nM of the Rev-mix1 primer (Table 1), 400 μM dNTP mix, 0.6 μL RiboLock RNase Inhibitor, 1 μL OneStep Enzyme Mix, and 5 μL of the purified nucleic acid. The cycling protocol was as follows: RT-step at 50 °C for 30 min and 95 °C for 15 min; 10 cycles of denaturation at 94 °C for 20 s, primer annealing at 62–53 °C for 30 s with 1 °C decrease per cycle, and extension at 72 °C for 1 min; 30 cycles of denaturation at 95 °C for 30 s, primer annealing at 53 °C for 30 s, and extension at 72 °C for 1 min; a final extension step at 72 °C for 10 min. Following one-step RT-PCR, the second PCR of the nested protocol corresponded to that described for other PCRs using DreamTaq DNA Polymerase (Thermo Fisher Scientific, Waltham, MA, USA).

PCR products of ~600 bp in length were purified from agarose gel with the NucleoSpin Gel and PCR Clean-Up Kit (Macherey-Nagel, Düren, Germany), and Sanger sequencing was performed for sequence confirmation by a service company.

The novel oligonucleotides were compared to previously published primers using nested PCR amplification set to that of those described in the referred protocol, using SSIV Reverse Transcriptase (Thermo Scientific, Waltham, MA, USA) and DreamTaq DNA Polymerase (Thermo Fisher Scientific, Waltham, MA, USA) or QIAGEN OneStep RT-PCR Kit (QIAGEN, Hilden, Germany) and DreamTaq DNA Polymerase (Thermo Fisher Scientific, Waltham, MA, USA) in combination.

Pan-CoV PCRs were performed from 121 pooled swine samples following the RT step carried out with SSIV (Thermo Scientific, Waltham, MA, USA), as described above. The nested PCR primers developed in this study were applied according to the touchdown protocol, also detailed in Section 2.3.

### 2.3. In Vitro Transcription

RNA standards were generated by in vitro transcription using the TranscriptAid T7 High Yield Transcription Kit (Thermo Fisher Scientific, Waltham, MA, USA) according to the manufacturer’s instructions. The T7 promoter sequence was incorporated into the linear DNA templates through the forward oligonucleotides Fw-T7-mix, while the reverse primers of the Rev-T7-mix contained an extension on the 5′ ends (Table 1). Following transcription, the ~793 bp long RNA standards were purified with the NucleoSpin RNA Virus kit (Macherey-Nagel, Düren, Germany) and were stored at −70 °C in nuclease-free water supplemented with RiboLock RNase Inhibitor (Thermo Fisher Scientific, Waltham, MA, USA). The concentration of the RNA standards was measured with a NanoDrop spectrophotometer (Thermo Fisher Scientific, Waltham, MA, USA), and a tenfold serial dilution of the RNAs was prepared in nuclease-free water containing RNase inhibitor. The copy number was set to 5 × 10^4^–5 × 10^0^ RNA copies per PCR reaction.

### 2.4. Software

Coronavirus reference sequences were selected based on the literature and retrieved from GenBank for primer design. The nt alignments of RdRp sequences were generated with the MAFFT algorithm of Geneious Prime v.2024.0.3 (Biomatters, Auckland, New Zealand). The generated sequences were checked with the Basic Local Alignment Search Tool (BLAST, online platform) [25]. Phylogenetic analyses were executed with the MEGA X software, neighbor-joining method, and p-distance model, using 1000 bootstrap replicates [26].

## 3. Results

As expected, based on the sequence alignment, the RdRp gene proved to be the most appropriate target for broad-spectrum PCR amplification. The primary oligonucleotide tests and in vitro transcription were executed using clinical samples and CoV isolates of human (α-CoV: NL-63; β-CoV: SARS-CoV, SARS-CoV-2 variants “alpha”, “delta”, and “omicron”, and OC43 isolates) and animal origin (α-CoV: FCoV isolated from a cat with feline infectious peritonitis, CCoV, PEDV, TGEV, and bat CoVs; γ-CoV: IBV). Unfortunately, specimens containing δ-CoV were unavailable. Where available, the oligonucleotides were tested with more than one specimen for the respective coronavirus (bat CoVs, CCoV, PEDV, TGEV, IBV, and SARS-CoV-2 variants).

A large number of degenerate oligonucleotides were examined with a basic protocol involving SSIV reverse transcriptase and DreamTaq DNA polymerase. Further optimization was carried out using the most suitable forward and reverse primer mixes. RT was performed with both random hexamer and CoV-specific reverse primer sets. The annealing temperature was investigated in matrices, and the oligonucleotides tolerated a broad spectrum of temperatures. A temperature of 53 °C was selected for annealing in subsequent experiments. Finally, following random hexamer RT priming, a primer combination suitable for semi-nested PCR produced the most robust results, containing one forward and two reverse primer sets (Fw-mix, Rev-mix1, and Rev-mix2, Table 1). The first PCR amplified a fragment of the coronavirus RdRp that was approximately 760 bp long, while the second, nested PCR product was approximately 600 bp in length. Although the amplicons of the first PCR could be visualized on an agarose gel, the bands were less intense than those of the second, nested PCR.

During the optimization of the pan-CoV semi-nested PCR with the chosen primer sets, the suitability of the previously defined factors (such as primer concentrations, annealing temperature, or the amplification cycles) was also continuously reviewed. Amplification was efficient with 30–45 cycles for the first, and 35–45 cycles for the nested PCR. While both methods were effective, random hexamers were more suitable for RT priming than Rev-mix1.

To determine the limit of detection (LOD) of the RT-PCR, RNA standards were generated by in vitro transcription. After measuring the quality and quantity, tenfold serial dilutions were prepared from the RNAs, with 5 × 10^4^–5 × 10^0^ RNA copies/reaction. The RNA series were investigated in triplicate, and the assay was repeated a minimum of three times. Dilutions of the in vitro transcribed RNAs were prepared both with and without RNase inhibitor. The LOD values were far better (one or two orders of magnitude) when the inhibitor enzyme was used, even though the RNAs were always freshly diluted with nuclease-free water and plastic labware.

**Figure 1:**
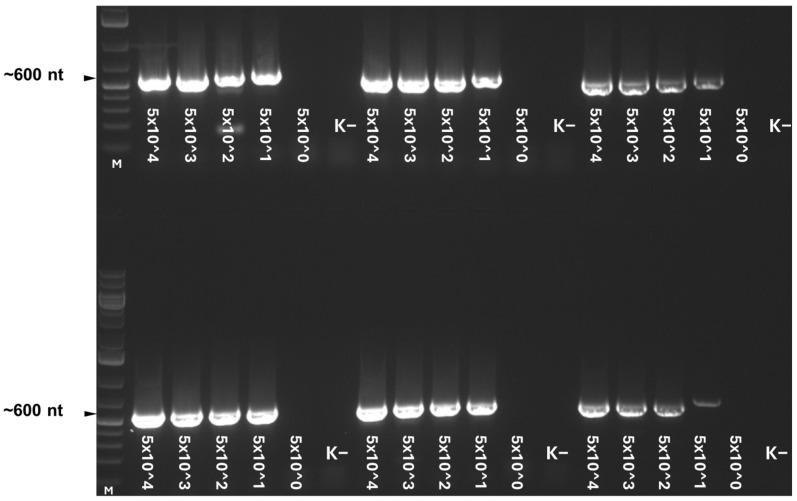
Agarose gel electrophoresis of products amplified by the novel coronavirus-specific broad-spectrum semi-nested RT-PCR from in vitro transcribed feline coronavirus RNA-dependent RNA polymerase fragments (six repeats from two RNA dilution series). Reverse transcription was implemented using random hexamer priming. The first PCR of the semi-nested amplification reaction was carried out with a touchdown cycling protocol. The values represent the limit of detection expressed as copies/reactions. M: DNA ladder.

RT was performed using two RT enzymes (SSIV and RevertAid Reverse Transcriptase) and random hexamers, as well as with one-step RT-PCR (QIAGEN OneStep RT-PCR Kit) and the Rev-mix1 oligonucleotide set. The RT step was followed by the semi-nested PCR, while a single nested PCR was carried out following the one-step RT-PCR. In addition to the standard cycling protocol with annealing at 53 °C, touchdown cycling was assessed. In most cases, the lowest LOD values were obtained using the SSIV RT enzyme and random hexamers in combination with the novel primer sets and the touchdown cycling protocol in the first PCR, slightly lower than the LOD values measured for the one-step RT-PCR protocol (Figure 1, Table 2). The LODs gained by the use of the RevertAid RT enzyme were orders of magnitude higher than the results obtained by the other two RT enzymes, the SSIV and the OneStep RT-PCR enzyme (Table 2).

**Figure 2:**
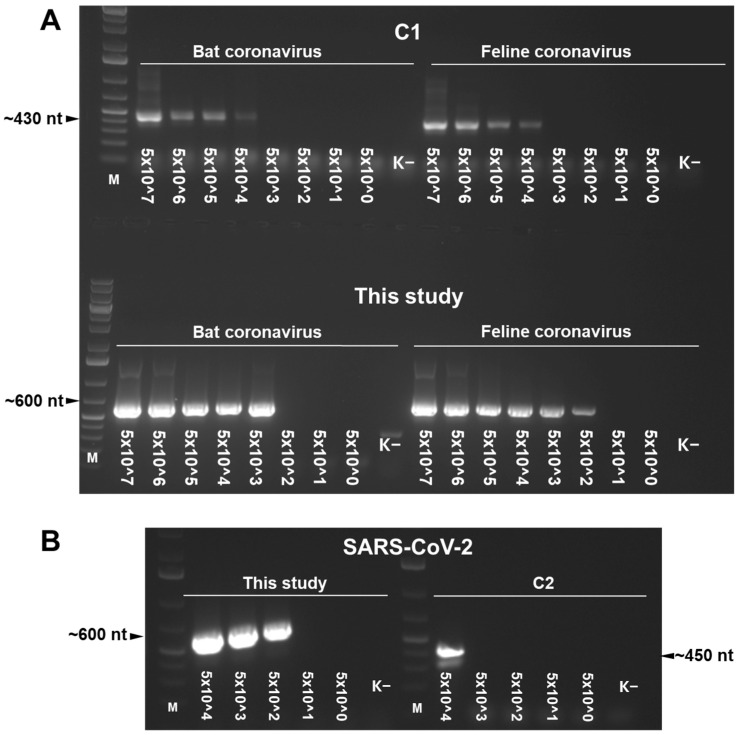
Agarose gel electrophoresis of products amplified by coronavirus-specific broad-spectrum oligonucleotides published previously (panel (**A**): primer set C1; panel (**B**): primer set C2) or developed in the present study. In every comparison, the amplification protocol was set to that described for reference primers C1 and C2, and it was also applied to the novel primer sets. The values represent the limit of detection expressed as copies/reactions, obtained for in vitro transcribed bat and feline coronavirus (panel (**A**)), and SARS-CoV-2 (panel (**B**)) RNA-dependent RNA polymerase fragments. M: DNA ladder.

The novel oligonucleotides were compared with two other broad-spectrum CoV-specific primer sets (C1 and C2) described earlier in relevant publications, applying the referred RT enzymes and cycling protocols, also for primers Fw-mix, Rev-mix1, and Rev-mix2. In general, the newly designed primers resulted in lower LOD values and higher specificity (the previously published primers did not present positivity with all RNA standards) and produced more intense bands in the agarose gel than the other two primer sets (Figure 2).

To evaluate its effectiveness, the PCR system developed here was used to test 121 pooled swine samples, and CoV sequences were identified in 24 (19.8%) (Table 3). Although RdRp fragments of multiple coronaviruses could be amplified in the same reaction, Sanger sequencing identified one virus in each sample (GenBank acc. no. PV9488443-PV9488453 and PX677392-PX677399). Twelve of the sequences showed the highest identity with PRCV (99.64% nt identity), and twelve with PHEV (99.10% nt identity) sequences deposited in GenBank. Two nasal swab samples tested positive for PRCV, four for PHEV, while the other eighteen were oral fluid specimens, ten PRCV, along with eight PHEV sequences. The identified coronaviruses were found at five different settlements/farms in Hungary, with two representing both PRCV and PHEV (labeled with the letters ST and KB) (Table 3).

**Figure 3:**
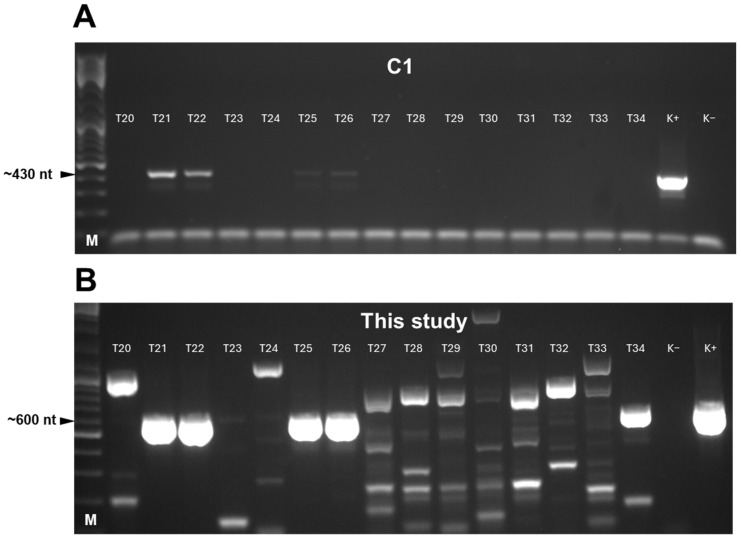
Representation of agarose gel electrophoresis of PCR products amplified by coronavirus-specific broad-spectrum oligonucleotides published previously (panel (**A**): primer set C1, 20 μL of PCR mixture) or developed in the present study (panel (**B**): 10 μL of PCR mixture). In every comparison, the amplification protocol was set to that described for the reference primer set C1. M: DNA ladder.

As a comparison, the swine field samples were investigated with one of the previously published pan-CoV-nested PCRs (C1 primer set), showing better results with the applied ten in vitro transcribed reference RNAs. In total, 8 of the 121 specimens represented CoV sequences with the C1 primer set that were also detected with the novel oligonucleotides (Table 3; Figure 3). Six amplicons were amplified from PRCV, while two were amplified from PHEV genomes.

**Figure 4:**
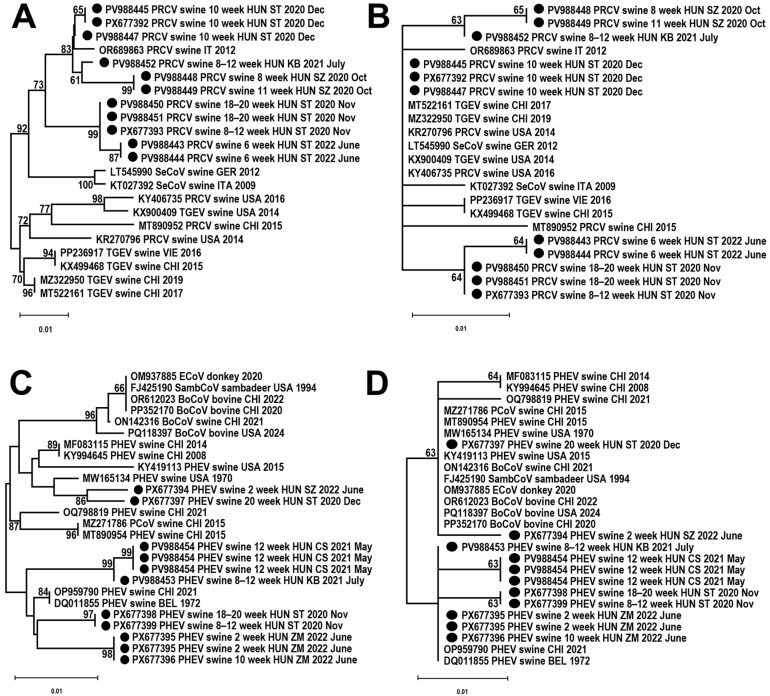
Unrooted neighbor-joining phylogenetic trees of partial RNA-dependent RNA polymerase nucleotide (nt) and amino acid (aa) sequences generated from the genome of porcine respiratory coronavirus (PRCV, panel (**A**)—nt sequences; panel (**B**)—aa sequences) and porcine hemagglutinating encephalomyelitis virus (PHEV, panel (**C**)—nt sequences; panel (**D**)—aa sequences) identified in swine samples in Hungary (highlighted with black circles).

Weak band intensity was detected in the agarose gels for further samples tested with both PCR systems. However, due to artifacts generated by the use of degenerated primers, these were not considered CoV-positive results without the opportunity of direct sequencing of the low-concentration products (for example, T25 and T26 samples, panel A; T23 sample, panel B). The determined partial PRCV and PHEV nt and aa sequences showed slight differences, even those originating from the same farm (ST) but from pigs of variable ages or from specimens collected at different dates (Figure 4).

## 4. Discussion

The explosion in the number of CoVs described and their importance in human and animal health led to an increased need to survey these viruses. Metagenomics is a proper tool for sequence-independent identification and comprehensive survey of viral genome diversity, but it is not an accessible and ideal method for many laboratories due to the costs and time-consuming processing. Therefore, PCR could be a simpler and, in many cases, more specific option for broad-spectrum detection of pathogens. Of course, in each case, the tools should be chosen after considering the goal of a study and the available budget.

The most common method used for the diagnosis of specific CoVs is qPCR-based amplification of S-encoding genomic fragments, while, as our research confirmed, RdRp is the optimal target for broad-spectrum PCR detection. Although most pan-CoV PCR systems are appropriate for the surveillance of mammalian viruses, few also recognize avian CoVs, such as IBV [27,28,29,30]. The primer sets designed here were developed to identify variable coronaviruses of birds as well, with a relatively long amplicon size. The forward primers targeted a commonly used, conserved region of the CoV RdRp [27,28,30,31,32], while most of the potential reverse priming positions represented higher sequence variability; thus, they had to be carefully selected. The Rev1 primers were designed for a position that has not yet been used in the publications we have studied. One primer for a non-nested PCR was found that matched the location of the Rev2 primers [30]. To cover most of the RdRp sequence types, we designed more than one degenerate primer into a set for a given priming position to account for the variability found in the reference sequences.

The choice of nucleic acid standards for optimization and comparisons is a crucial issue. Application of DNA standards, such as PCR products, cDNA, synthesized DNA, and plasmids, could result in lower LOD values for RNA viruses [27,30,32]. In contrast to the most commonly utilized DNA templates, in vitro transcribed RNAs were produced to measure the LOD that could better represent RT-PCR of an RNA virus sequence [28]. Some studies have applied cultured CoVs and assumed the sensitivity and LOD based on TCID50, PFU, and HA titers [29]. Unfortunately, the propagation of numerous CoVs remains unresolved; thus, the number of viruses that can be tested this way is limited. Additionally, it is important to note that despite calculations involving infectious virions, PCR amplifies all target RNAs, including the non-encapsidated molecules as well.

To reveal the importance of enzyme usage, we tested three different RT enzymes together with random and specific primers, in normal and touchdown PCR cycling protocols on RNA standards. There was a significant difference between the efficiency of the two-step RT enzymes. The more robust and thermostable SSIV enzyme with random hexamer priming resulted in the lowest LOD values. Furthermore, touchdown PCR cycling proved to be the most suitable for annealing of the degenerated, broad-spectrum primers. In contrast to the specific RT priming, the advantage of random hexamer usage could also be exploited for the detection of co-infections of other RNA viruses, without preparation of new RT reactions.

Due to different standards and amplification conditions, the LOD values reported in publications are difficult to compare. Nevertheless, the 5–50-copy LOD and the robustness of the bands achieved in this study are promising. However, as our results showed, selecting the appropriate RT enzyme and preventing nucleic acid degradation with an RNase inhibitor greatly affect the success of virus identification.

To evaluate its suitability, surveillance of CoVs was conducted using the newly established RT-PCR system on swine samples. PRCV is a variant of TGEV with a deletion in the ORF S and an alteration in the ORF3 sequence, as well as scattered with point mutations [5,33]. The novel oligonucleotide system could detect TGEV; thus, its ability to identify PRCV was less unexpected. Due to the similarity in the RdRp sequence, distinguishing between TGEV and PRCV is hardly possible. Furthermore, in general, recombination and deletion mutations are frequent events among porcine CoVs; thus, whole-genome sequencing should be performed to identify and characterize the viruses found. The established RT-PCR was not tested with PRCV and PHEV RNA, but the results indicate its suitability for the detection of at least four swine CoVs.

In Hungary, infections with PEDV, TGEV, and PRCV have been previously revealed [34,35,36,37,38]. TGEV and PRCV have been found in cases with gross pathological lesions of weight loss and dehydration and with mild villous atrophy during histopathology [34]. PEDV was first identified in 2016 in piglets with diarrhea and vomiting, causing increased mortality at a farrow-to-finish farm [35]. Subsequently, further PEDV strains have been described, showing similarities to those from Slovenia, a neighboring country of Hungary [37]. Broad-spectrum coronavirus screening has not been performed previously in healthy animals in this country. In this study, oral fluid and nasal samples of clinically healthy swine were examined with a newly established RT-PCR system to discover CoV circulation in the herds investigated. PRCV (PRCV/TGEV) and PHEV were found in all investigated age groups, suggesting that these viruses circulate at these farms without provoking any disease. PEDV was not detected at any locations. Identification of PRCV and PHEV sequences in these types of specimens is consistent with data suggesting that these viruses primarily infect cells of the respiratory (PRCV/PHEV) or gastrointestinal tract (TGEV) [5]. While TGEV is an enteral pathogen, PRCV is particularly associated with respiratory diseases [5]. However, stool specimens were not examined in this study. PHEV is characterized as the only neurotropic CoV belonging to the *Betacoronavirus* genus. Following infection of the initial site, the virus disseminates to the central nervous system. PHEV infection in swine is accompanied by influenza-like symptoms, vomiting, and wasting disease, as well as encephalomyelitis [39,40,41]. These viruses can be devastating for piglets, but they are often found in subclinical infections, as in the herds of the presented survey.

Owing to the use of degenerated primers in broad-spectrum PCR systems, artifacts may be amplified, along with specific PCR products that are separated during gel electrophoresis. The presented PCR system contains highly degenerated oligonucleotides, which, as a limitation, implies these consequences. Therefore, verification is recommended and also needed for specific virus identification via sequencing in cases of such systems, as was carried out. Although direct sequencing was applied, multiple templates could be amplified, requiring other tools for sequence determination, such as high-throughput amplicon sequencing, which is planned to be introduced in our forthcoming studies as well. Following RT-PCR and preliminary sequence analysis for estimation of CoVs and their host spectra, complete genome sequencing can be attempted for precise classification of the viruses identified and to reveal their sequence diversity.

The novel detection system is promising, but its extensive application to field samples will reveal its true utility instead of the use of RNA templates. Although genomic sequences of some CoVs of the three genera were used as standards and could be amplified, we did not have the opportunity to test the PCR with deltacoronaviruses. However, even though a few representatives are examined, it is still unclear how effective the system is in the case of other members of a genus due to the diversity of CoVs. Primer sets require constant updating to ensure the widest possible recognition of viral sequences, which could be achieved with consideration of newly described viral sequences and additional virus strains available for testing in the future. Depending on the goal, simultaneous use of more than one RT-PCR system applying primers with distinct annealing sites could also be advantageous when dealing with highly variable pathogens. Due to the limitations encountered, the primers presented are not suitable for diagnostic purposes. However, by reducing the number of degenerated sites, following extensive optimization, these may be modified to detect a narrower spectrum of CoVs that are more closely related. Overall, the broad-spectrum RT-PCR offers the possibility of widespread screening for CoVs, obtaining data that can establish further studies.

## 5. Conclusions

The specificity and sensitivity of PCR-based broad-spectrum CoV detection systems vary. In addition, they require continuous review and adaptation to newly described coronaviruses. In this study, a pan-CoV PCR system was established that is suitable for the identification of human and animal coronaviruses. Compared to two other systems, the new primer set performed better. The study revealed that enzyme usage highly influenced the limit of detection regarding the copy number.

Detection systems developed for a certain pathogenic coronavirus are more sensitive due to their specific properties. The assay described is primarily suitable for research purposes and can contribute to the assessment of the host spectrum and diversity of coronaviruses.

## Figures and Tables

**Table 1:** The oligonucleotide sequences described in this study.

	Primer Set	Primer Sequence 5′–3′
Semi-nested PCR	Fw-mix	GGNTGGGAYTAYCCNAAATGTGA
		GGNTGGGAYTAYCCNAAGTGCGA
		GGNTGGGAYTAYCCNAAGTGTGA
	Rev-mix1	ARNGGRTANGCRTCWATTGC
		ARNGGRTANGCRTCWATGGC
		ARNGGRTANGCRTCWATAGC
	Rev-mix2	TGYTGNGARCARAAYTCRTGNGGTCC
		TGYTGNGARCARAAYTCRTGNGGACC
		TGYTGNGARCARAAYTCRTGNGGGCC
		TGYTGNGARCARAAYTCRTGNGGCCC
in vitro transcription	Fw-T7-mix	TAATACGACTCACTATAGGGGGNTGGGAYTAYCCNAAATGTGA
		TAATACGACTCACTATAGGGGGNTGGGAYTAYCCNAAGTGCGA
		TAATACGACTCACTATAGGGGGNTGGGAYTAYCCNAAGTGTGA
	Rev-T7-mix	TCCTCCTCCTCCARNGGRTANGCRTCWATTGC
		TCCTCCTCCTCCARNGGRTANGCRTCWATGGC
		TCCTCCTCCTCCARNGGRTANGCRTCWATAGC

**Table 2:** Limit of detection values (copies/reaction) measured for the novel coronavirus-specific primers using variable reverse transcriptase and touchdown PCR profiles.

	SuperScript IV RT	QIAGEN One-Step RT-PCR	RevertAid RT
SARS-CoV	5 × 10^1^	5 × 10^2^	5 × 10^2^
SARS-CoV-2	5 × 10^1^	5 × 10^2^	5 × 10^2^
OC43	5 × 10^0^	5 × 10^1^	5 × 10^3^
NL-63	5 × 10^1^	5 × 10^2^	5 × 10^2^
PEDV	5 × 10^0^	5 × 10^1^	5 × 10^2^
TGEV	5 × 10^1^	5 × 10^2^	5 × 10^1^
CCoV	5 × 10^0^	5 × 10^1^	5 × 10^2^
FCoV	5 × 10^1^	5 × 10^1^	5 × 10^1^
Bat	5 × 10^1^	5 × 10^2^	5 × 10^1^
IBV	5 × 10^0^	5 × 10^2^	5 × 10^3^

**Table 3:** Results of coronavirus surveillance performed with the novel oligonucleotides and protocol (this study), and a previously published (C1) pan-coronavirus-nested PCR on swine samples, representing the samples that tested positive.

Sample Type	Positive by Primers of This Study	Positive by Primers C1	Age of Pigs (Week Old)	Sampling Date	Sampling Site (Farm)	GenBank Acc. No.
Porcine respiratory coronavirus						
Oral fluid	1	-	8	October 2020	SZ	PV988448
Oral fluid	1	-	11	October 2020	SZ	PV988449
Oral fluid	1	-	8–12	November 2020	ST	PX677393
Oral fluid	2	2	18–20	November 2020	ST	PV988450-51
Oral fluid	4	2	10	December 2020	ST	PV988445-47, PX677392
Oral fluid	1	-	8–12	July 2021	KB	PV988452
Nasal swab	2	2	6	June 2022	ST	PV988443-44
Porcine hemagglutinating encephalomyelitis virus						
Oral fluid	1	-	8–12	November 2020	ST	PX677397
Oral fluid	1	-	18–20	November 2020	ST	PX677398
Oral fluid	1	-	20	December 2020	ST	PX677399
Oral fluid	4	1	12	May 2021	CS	PV988454
Oral fluid	1	1	8–12	July 2021	KB	PV988453
Nasal swab	1	-	2	June 2022	SZ	PX677394
Nasal swab	2	-	2	June 2022	ZM	PX677395
Nasal swab	1	-	10	June 2022	ZM	PX677396

## Data Availability

The authors confirm that the data supporting the findings of this study are available within the article and Supplementary Material File S1. The partial genome sequences have been deposited in the GenBank database with accession numbers (GenBank acc. nos. PV9488443-PV9488453 and PX677392-PX677399).

## Supplementary Materials

The following supporting information can be downloaded at https://www.mdpi.com/article/10.3390/ani16030358/s1: File S1: The primers and coronavirus RNA-dependent RNA polymerase sequences used for primer design.
